# Daily Use of Extra Virgin Olive Oil with High Oleocanthal Concentration Reduced Body Weight, Waist Circumference, Alanine Transaminase, Inflammatory Cytokines and Hepatic Steatosis in Subjects with the Metabolic Syndrome: A 2-Month Intervention Study

**DOI:** 10.3390/metabo10100392

**Published:** 2020-10-02

**Authors:** Angelo M. Patti, Giuseppe Carruba, Arrigo F. G. Cicero, Maciej Banach, Dragana Nikolic, Rosaria V. Giglio, Antonino Terranova, Maurizio Soresi, Lydia Giannitrapani, Giuseppe Montalto, Anca Pantea Stoian, Yajnavalka Banerjee, Ali A. Rizvi, Peter P. Toth, Manfredi Rizzo

**Affiliations:** 1 Department of Health Promotion Sciences Maternal and Infantile Care, Internal Medicine and Medical Specialties (PROMISE), University of Palermo, 90127 Palermo, Italy; pattiangelomaria@gmail.com (A.M.P.); rosaria.vincenza.giglio@alice.it (R.V.G.); toninoterranova@unipa.it (A.T.); maurizio.soresi@unipa.it (M.S.); lydiagiannitp@gmail.com (L.G.); giuseppe.montalto@unipa.it (G.M.); manfredi.rizzo@unipa.it (M.R.); 2 Division of Research and Internationalization, ARNAS-Civico Di Cristina e Benfratelli Hospital, 90127 Palermo, Italy; giuseppe.carruba@arnascivico.it; 3 Medical and Surgical Sciences Department, University of Bologna, 40138 Bologna, Italy; arrigo.cicero@unibo.it; 4 Department of Hypertension, Chair of Nephrology and Hypertension, Medical University of Lodz, 90-419 Lodz, Poland; Maciej.banach@iczmp.edu.pl; 5 Polish Mother’s Memorial Hospital Research Institute (PMMHRI) in Lodz, 93-338 Lodz, Poland; 6 Department of Diabetes, Nutrition and Metabolic Diseases, Carol Davila University of Medicine and Pharmacy, 050474 Bucharest, Romania; ancastoian@yahoo.com; 7 Mohammed Bin Rashid University of Medicine and Health Sciences, 505055 Dubai, UAE; Yajnavalka.Banerjee@mbru.ac.ae; 8 Division of Endocrinology, Diabetes and Metabolism, University of South Carolina School of Medicine, Columbia, SC 29203, USA; ali.abbas.rizvi@emory.edu; 9 Division of Endocrinology, Metabolism, and Lipids, Emory University School of Medicine, Atlanta, GA 30322, USA; 10 CGH Medical Center, Sterling, IL 61081, USA; peter.toth@cghmc.com; 11 School of Medicine, University of Illinois, Peoria, IL 60612, USA; 12 Johns Hopkins University School of Medicine, Baltimore, MD 21205, USA

**Keywords:** metabolic syndrome, olive oil, oleocanthal, polyphenols, cytokines

## Abstract

Extra virgin olive oil (EVOO) intake is associated with reduced cardiovascular risk, and its phenolic compound oleocanthal (OC) has anti-oxidant and anti-inflammatory properties. The cardiometabolic effects of EVOO with a high OC concentration have not been fully elucidated. We administered EVOO with a high OC concentration daily to 23 subjects with the metabolic syndrome (MetS) and hepatic steatosis (15 men and 8 women, age: 60 ± 11 years) for 2 months. Anthropometric data, metabolic parameters, hepatic steatosis (by fatty liver index, FLI), abdominal fat distribution (by ultrasound), and pro- and anti-inflammatory cytokines were assessed before and after the intervention. EVOO supplementation was associated with a reduction in body weight, waist circumference, body mass index (BMI), alanine transaminase and FLI, as well as interleukin (IL)-6, IL-17A, tumor necrosis factor-α and IL-1B, while IL-10 increased. Maximum subcutaneous fat thickness (SFT max) also increased, with a concomitant decrease in the ratio of visceral fat layer thickness/SFT max. Correlation analysis revealed positive associations between changes in body weight and BMI and those in SFT max, along with an inverse association between changes in IL-6 and those in SFT max. In conclusion, ingestion of EVOO with a high OC concentration had beneficial effects on metabolic parameters, inflammatory cytokines and abdominal fat distribution in MetS subjects with hepatic steatosis, a category of patients at high cardiometabolic risk.

## 1. Introduction

The relationship between the traditional Mediterranean diet (MD) and human health has been known since ancient times and several studies have shown the beneficial effects of the MD in preventing diabetes and cardiovascular diseases (CVD) [1,2,3], as well as those on weight control, without significant adverse events [4,5,6]. Extra virgin olive oil (EVOO) is a major component in the MD [7], being recognized as one of the healthiest products originating from the Mediterranean region [7]. The beneficial properties belonging to EVOO have been ascribed to its components, especially to the phenolic constituents [8,9,10,11]. Oleocanthal (OC) is a phenolic compound found mostly in EVOO which is responsible for its bitter taste, having strong anti-inflammatory properties based on the inhibition of cyclooxygenase (COX) [12]. For this reason, OC is recognized as a natural non-steroidal anti-inflammatory agent [13,14,15,16]. 

Several studies have shown that the phenolic compounds of EVOO have beneficial anti-inflammatory, anti-microbial and anti-oxidant activities [17]. It has been also suggested that long-term daily intake of OC-containing EVOO may be, at least in part, responsible for the health protection of Mediterranean populations [12]. Yet, the metabolic syndrome (MetS) is rapidly increasing in such populations, since dietary habits and lifestyles have strongly changed in the last decades [18,19]. A growing number of studies indicate that nutraceuticals have several benefits on more than one component of the MetS [20,21,22,23,24,25]. In addition, nonalcoholic fatty liver disease is one of the emerging components of the MetS [26,27,28]. The presence of hepatic steatosis in MetS subjects is a negative prognostic factor for future cardiometabolic events and is associated with future events independently of other cardiovascular (CV) risk factors [29]. Increasing evidence suggests that nutraceuticals can have several benefits on hepatic parameters in MetS subjects [30], but the effects of EVOO and OC on MetS, with or without the presence of hepatic steatosis, are still unknown. 

The aim of this study was to evaluate the effects of daily use of EVOO rich in OC, on anthropometric, metabolic and inflammatory parameters, as well as on abdominal fat distribution, in subjects with the MetS and hepatic steatosis, which represents a category of patients at high risk for developing future cardiometabolic events.

## 2. Results

We found that 2 months of EVOO supplementation led to a significant reduction in body weight, body mass index (BMI), waist circumference, alanine transaminases (ALT) and fatty liver index (FLI) in MetS patients with hepatic steatosis (Table 1).

In addition, we tested if there were any gender-specific changes in the cardiometabolic effects of EVOO after the supplementation, although the number of women was low. In men, only waist circumference, and in women, only body weight significantly changed after 2 months (data not shown). However, when comparing the two groups, the differences between genders did not reach statistical significance for any parameter.

In addition, levels of all the measured pro-inflammatory cytokines (IL (interleukin)-6, IL-17A, TNF-α, IL-1B) decreased significantly, while concentrations of the anti-inflammatory cytokine IL-10 significantly increased (Table 2). 

Regarding abdominal fat distribution, we found that the maximum subcutaneous fat thickness (SFT max) significantly increased after olive oil supplementation, with a concomitant reduction in the ratio of visceral fat layer thickness/max. subcutaneous fat thickness (Table 3).

We also performed correlation analysis in order to assess potential associations between changes in all evaluated parameters (data not shown). There was a significant association between changes in both body weight and BMI and those in SFT max (r = 0.426, *p* = 0.043; r = 0.490, *p* = 0.018, respectively) and between changes in both body weight and BMI and those in the minimum pre-peritoneal fat thickness (PFT min) (r = 0.561, *p* = 0.012; r = 0.525, *p* = 0.021, respectively). In addition, we found a significant association between changes in IL-6 and those in SFT max (r = −0.505, *p* = 0.014) and between changes in IL-1B and those in PFT max (r = 0.662, *p* = 0.026).

## 3. Discussion

It is well known that the MD represents a dietary pattern characterized by the presence of different varieties of whole cereals, fresh fruits and vegetables, wheat products (bread, pasta), legumes, potatoes, beans, nuts and seeds, milk products (such as cheese and yogurt), blue fish and poultry consumed in moderate quantities, and a low consumption of red meat, refined sugar and animal fat. However, one of the main distinctive components of the MD is EVOO, representing the main source of dietary fat and polyphenols [31,32], the latter being potent inhibitors of reactive oxygen species and associated with a reduced risk for CVD and several types of cancer [2,33,34,35,36]. Several studies confirmed the beneficial effects of polyphenols in olive oil on heart disease risk factors and oxidative damage [37,38,39,40]. In addition, EVOO has been widely studied for its favorable effects on plasma concentrations of low-density lipoprotein cholesterol (LDL-C) and total cholesterol [33,39,41,42,43], as well as on blood pressure [43,44,45]. 

Owen et al. [46] suggested that the antioxidant phenolic fraction, along with high contents of squalene and oleic acid, may confer to olive oil its health-promoting properties. In this context, the Epicor study, investigating the association between consumption of fruits, vegetables, and olive oil and the incidence of coronary heart disease (CHD), has shown that the consumption of olive oil in the diet leads to a reduction in CVD risk in middle-aged Italian women without a diagnosis of stroke, myocardial infarction, diabetes or hyperlipidemia [47]. EVOO is able to counteract postprandial oxidative stress through down-regulation of nicotinamide adenine dinucleotide phosphate oxidase (NOX2) [48] and reduces the risk of diabetes among people at a high CVD risk [20,49,50]. The Primary Prevention of Cardiovascular Disease with Mediterranean Diet (PREDIMED) study, with a total of 7447 persons enrolled [51], has also shown that the addition of EVOO to an MD reduces the risk the incidence of major CV events. 

There are several studies that have considered the effects of other polyphenols derived from EVOO, but none have evaluated the clinical effects of EVOO with a high concentration of its phenolic compound OC. Previous studies have reported OC activity as a potent antioxidant and as a non-steroidal anti-inflammatory agent [12]. In fact, Beauchamp et al. [12] showed that the structure of OC and that of the anti-inflammatory drug ibuprofen share a similar molecular geometry and, like ibuprofen, OC exhibits COX-inhibiting activity. Furthermore, consumption of 9 mg of OC per day mediates an anti-inflammatory effect, which is equivalent to that achieved by 90 mg of ibuprofen. MetS is a constellation of disturbances detrimentally affecting the metabolic homeostasis of an individual, where metabolic overload induces stress reactions, such as oxidative, inflammatory, organelle and cell hypertrophy, leading to a vicious cycle where environmental, genetic and psychosocial factors interact through complex networks [52]. The difficulty in the management of MetS is a consequence of its multifactorial nature, where environmental, genetic and psychosocial factors interact through intricate networks, although inflammation is one of the key underlying factors spurring the progression of MetS and is directly linked to the adverse CV outcome [53,54]. Therefore, it is essential to assess novel anti-inflammatory strategies, especially in subjects with the MetS, for comprehensive cardiometabolic prevention [55,56,57].

In the present study, we have assessed the impact of EVOO with a high percentage of OC on metabolic parameters and abdominal fat distribution, as well as on pro- and anti-inflammatory cytokines, in adults with the MetS and hepatic steatosis, a population known to be at high risk for atherosclerotic CVD [58]. To the best of our knowledge, the present study is the first one to determine the beneficial effects of EVOO with a high percentage of OC in such high-risk subjects. We found that the consumption of high-OC EVOO beneficially affected the anthropometric parameters evaluated, with a reduction in body weight, BMI and waist circumference. Furthermore, although subjects did not change their lifestyle or eating habits, 2 months of dietary intervention with olive oil were enough to significantly reduce those parameters. The observed effects may be due to increased postprandial fat oxidation, which has been observed after a meal rich in olive oil [59]. On the other hand, a recent study showed that an MD enriched with EVOO can be an effective alternative option to low-fat diets for weight maintenance regimes in older overweight or obese adults [60].

We did not find, in the present study, any statistically significant variation in plasma lipids and glycemic parameters, such as fasting glycemia and glycated hemoglobin (HbA1c). Similarly, in a randomized placebo-controlled trial including 41 overweight and obese adults, the substitution of commonly used oil with EVOO for 3 months did change glycaemia and plasma lipids [61]. However, the absence of significant changes in these parameters may also be due to the relative short duration time and sample size of the study. Indeed, in a crossover study including 200 healthy male volunteers who were randomly assigned to three sequences of daily administration of 25 mL of three different olive oils (with a low, medium or high phenolic content), benefits on plasma lipids have been found [62]. Of interest, in the present study, we observed that the supplementation of EVOO with a high OC concentration significantly reduced ALT in MetS patients with hepatic steatosis, and it is known that transaminase concentrations are associated with the degree of hepatic steatosis, as assessed by ultrasound [63]. Therefore, although not directly demonstrated, we cannot exclude that olive oil use led to an improved degree of hepatic steatosis, which is supported by improved FLI, in spite of the limited period of follow-up. Furthermore, the hepato-protective action of EVOO, has been demonstrated in animals [64] and later confirmed in humans [65,66], although the effect of OC on hepatic function is still unclear.

Regarding abdominal fat distribution, we found a significant increase in SFT max, with a decrease in the ratio of visceral fat layer thickness/SFT max. This finding may have important clinical implications since visceral adipose tissue (VAT) is considered to be more metabolically active than subcutaneous adipose tissue (SAT), having a different capacity to secrete adipokines/cytokines, hormones and immune molecules [67]. It is also well documented that an excess of VAT can be considered as a good marker of an altered cardiometabolic risk profile associated with increased CVD risk [68]. Correlation analysis revealed positive associations between changes in body weight and BMI and those in SFT max, highlighting the concomitant effects of the supplementation of EVOO with a high OC concentration on anthropometric parameters and abdominal fat distribution. 

It has been previously reported that in healthy subjects, serum lipid levels were associated with total body fat, but more specifically abdominal SAT, with an association between serum triglycerides and waist circumference; in addition, in that population, levels of inflammatory IL-18 cytokine were associated with small, dense LDL [69,70]. Indeed, the quality, rather than only the quantity, of LDL-C is strongly associated with CV risk, with atherogenic small, dense LDL being a novel marker of such risk [71,72]. The relationship between inflammation, oxidative stress and small, dense LDL is very strict [73], with the latter representing a main feature of the MetS [74], and, even more, a strong predictive marker for future CV and cerebrovascular events in MetS patients [75]. It would be, therefore, of clinical value to assess, in future studies, the effect of EVOO with a high content in OC on small, dense LDL as well as on HDL functionality, beyond plasma lipid levels [76].

In the present study we also found that levels of pro-inflammatory cytokines (IL-6, IL-17A, TNF-α and IL-1B) significantly decreased, while those of anti-inflammatory cytokine (IL-10) significantly increased. This data suggests that the consumption of EVOO with a high content of OC can have important anti-inflammatory effects, and it is consistent with the natural anti-inflammatory properties of OC [14,77,78]. It has been previously reported that one of the mechanisms underlying the anti-inflammatory properties of OC is the inhibition of the nuclear factor-κB (NF-kB) pathway [79], which in turn may not only reduce the levels of pro-inflammatory cytokines but also attenuate cortical cyclooxygenase-2 (COX-2) signaling [80]. However, further investigations are needed for a better understanding of this aspect. Interestingly, a preclinical study has shown that an EVOO-enriched diet had the best results on the musculoskeletal system in an osteoarthritis rat model, including decreased IL-6 expression [65]. On the other hand, an MD rich in high quality EVOO also significantly increases IL-10 levels [40]. By correlation analysis, we interestingly found an inverse association between changes in IL-6 and those in SFT max, which further highlights the concurrent effects of olive oil supplementation on anti-inflammatory cytokines and abdominal fat distribution.

Potential limitations of the present study include the small number of subjects included; however, this allowed us to monitor the study population closely ensuring the participants adhered to the desired regimen, even more since it was a different type of pasta to be consumed almost daily. Furthermore, our study was open-label and lacked a control (placebo) group; however, all patients, prior to the intake of EVOO with high OC, followed an MD with EVOO containing standard contents of OC; therefore, this somewhat overcame the need of a placebo control group, since the population included in the study acted as their own control. Lastly, our study had a relatively short intervention period with only two points of measurements (baseline and end of the follow-up period), which made us unable to look at the variables in the continuous way, a fact that should be addressed in future studies.

Nevertheless, this study has several strengths, including the mono-cultivar type of olive oil and the novelty of the study with regards to the design strategy, as well as the large number of biochemical and ultrasound parameters assessed. All measurements were assessed in a blinded manner, which means that the ultrasound operator did not have access to previous scans, and all the biochemical parameters were assessed in aliquots with blinded codes.

## 4. Materials and Methods 

### 4.1. Study Design

Mono-cultivar EVOO with a high OC content was supplied by Prof. Tiziano Caruso from the Department of Agriculture and Forestry Sciences, University of Palermo, Italy, and used for the purpose of this study only. All patients were referred to our Unit of Diabetes and Cardiovascular Prevention for a clinical evaluation and included in the study consequently. The olive oil was administered to 23 subjects with the MetS at a fixed dose of 4 large spoons daily (which corresponded to 32 g of EVOO) during their main meals, e.g., at lunch and dinner, for a period of 60 days. The study population prior to the intake of EVOO with high OC acted as their own control since they followed an MD with EVOO containing standard contents of OC. This is one of the strengths of the study, as it allowed us to monitor the different parameters for each patient closely, without the specific need of a separate control group. During the study, the dose of the EVOO was maintained unmodified, and no other type of oil was allowed. 

Inclusion criteria included: European descent following an MD, age between 18 and 70 years, the diagnosis of MetS as defined by international consensus [81] and the presence of hepatic steatosis (as assessed by abdominal ultrasound). Exclusion criteria included acute and chronic renal failure, liver diseases, acute illnesses, chronic inflammatory or infective diseases and tumors, as well as alcohol consumption (>30 g/day for men and >20 g/day for women). All subjects were followed up for 60 days, and a similar evaluation was performed at baseline and after the follow-up period, including a medical examination, biochemical analyses and an abdominal ultrasound. All concomitant therapies were kept constant during the study, and this was achieved in all patients due to the relatively short duration of the study. Notably, all patients did not use any natural supplement for dyslipidemia. The adopted procedures were in agreement with the Helsinki Declaration of 1975 as revised in 1983, and the study was approved by the Ethical Committee of the University of Palermo. 

All patients gave informed written consent to participate in the study and completed the “Epic” Food Frequency Questionnaire [82] in order to assess their eating habits and the frequency of consumption of foods and beverages. All patients were accustomed to Mediterranean dietary habits, since they were born and have been living all their life in the same metropolitan area in Italy. During the study, patients were strongly advised not to alter their dietary habits, nor change their lifestyle. In fact, this was ensured carefully by weekly follow-up telephone calls. In line, we followed a similar strategy [83] to ensure that the study population adhered to the desired regimen. Baseline characteristics of patients are shown in Table 4. A sedentary lifestyle with little physical activity was common among the subjects. 

### 4.2. Biochemical Analyses

All blood samples were taken after at least 10 h overnight fast. We measured by routine laboratory methods plasma lipids as well as serum aspartate transaminases (AST), ALT and gamma glutamyl-transferase (GGT). LDL-C was calculated using the Friedewald formula. FLI score was used as a marker of liver steatosis, with the following formula [84]: FLI = (e0.953 × loge (TG) + 0.139 × BMI + 0.718 × loge (GGT) + 0.053 × waist circumference − 15.745)/(1 + e0.953 × loge (TG) + 0.139 × BMI + 0.718 × loge (GGT) + 0.053 × waist circumference − 15.745) × 100.

### 4.3. Cytokines’ Analyses

Blood samples were collected in tubes without ethylenediamine tetraacetic acid (EDTA), and serum was separated from whole blood by low-speed centrifugation at 2500 rpm for 15 min at 4 °C. We analyzed four pro-inflammatory cytokines (IL-6, IL-17A, TNF-α, IL-1B) [85,86,87,88] and one anti-inflammatory cytokine (IL-10) [89] by Luminex assays, coupled to BioPlex Manager software [90].

### 4.4. Ultrasound Examination

Abdominal fat distribution was assessed by ultrasound in the morning, after fasting for at least 10 h, by a single investigator/physician (AT), in order to avoid the risk of measure-to-measure inter-investigator bias. A real-time Philips 5000 HDI apparatus with a 2–5 MHz convex multi-frequency probe and 5–12 MHz multi-frequency linear probe was used. All patients had hepatic steatosis, as an inclusion criteria for the study [91]. Steatosis severity was scored as: grade 1 (mild steatosis), characterized by increased echogenicity; grade 2 (moderate steatosis), accompanied by increased echogenicity and posterior beam attenuation with slightly impaired visualization of the intrahepatic vessels and diaphragm; grade 3 (severe steatosis), with a marked increase in echogenicity and marked posterior beam attenuation resulting in failure to demonstrate the intrahepatic vessels, diaphragm, and posterior right lobe of the liver [92]. All patients included in the present study had moderate steatosis. The SFT max was measured at 5 cm from the umbilicus on the xypho-umbilical line, whereas the minimum subcutaneous fat thickness was measured from the region just below the xyphoid process [93]. Such measurements were taken directly from the screen using the electronic calipers at the skin-fat (excluding skin) and fat-muscle interfaces, and then the mean thickness was calculated. Preperitoneal fat layer measurements were taken from the region where it is most clearly seen just below the xyphoid process, between the internal face of linea alba and surface of the liver. Measurement of preperitoneal fat at the upper and lower edges of the liver was avoided because the preperitoneal fat layer changes abruptly at these regions. A convex-array probe (3.5 MHz) was used for measuring the visceral fat layer thickness taken between the internal face of the abdominal muscle and the anterior wall of the aorta [94]. All patients were asked to hold their breath during the examination; special care was taken to keep the probe just touching the skin to prevent compression of the fat layers. 

## 5. Statistical Analysis

Statistical analyses were performed using SPSS software (V.17.0 for Windows, SPSS Inc., Chicago, IL, USA). All variables were tested for normality using the Kolmogorov–Smirnov test. Data are expressed as mean ± standard deviation for parametric variables, whereas categorical variables are expressed as percentages. Baseline characteristics were compared using the Chi-square test. We used the paired t-test for normally distributed parameters and Wilcoxon rank test for nonparametric variables, while the Spearman rank correlation method was used for the correlation analysis. 

## 6. Conclusions

To our knowledge, this is the first study investigating the effects of EVOO supplementation with a high concentration of OC in subjects with MetS and hepatic steatosis, a category of patients at high risk for developing future cardiometabolic events. Our findings suggest that such olive oil supplementation has multiple beneficial effects on anthropometric and biochemical parameters, including inflammatory cytokines and FLI as well as abdominal fat distribution. Further studies may provide a better understanding of mechanisms underlying the effects of OC on inflammation and, consequently, potential therapeutic approaches for the prevention and/or reduction in overall cardio-metabolic risk in such a high-risk population of MetS patients.

## Figures and Tables

**Table 1:** Effect of extra virgin olive oil (EVOO) with a high oleocanthal (OC) concentration on anthropometric and laboratory parameters (*n* = 23).

	Baseline	After 2 Months	*p*-Value
Body weight (kg)	87 ± 17	85 ± 16	0.035
BMI (kg/m^2^)	31 ± 4	30 ± 4	0.031
Waist circumference (cm)	108 ± 12	105 ± 10	0.037
Glycemia (mmol/L)	5.35 ± 1.11	5.86 ± 1.34	0.055
HbA1c (%)	6.13 ± 0.60	6.05 ± 0.45	0.192
Total cholesterol (mmol/L)	4.61 ± 0.98	4.60 ± 0.89	0.851
HDL-cholesterol (mmol/L)	1.17 ± 0.25	1.16 ± 0.25	0.392
LDL-cholesterol (mmol/L)	2.82 ± 0.72	2.72 ± 0.69	0.422
Triglycerides (mmol/L)	1.74 ± 0.75	1.71 ± 0.98	0.909
Aspartate transaminases (AST) (mU/mL)	29.21 ± 26.88	22.32 ± 7.52	0.167
Alanine transaminases (ALT) (mU/mL)	38.52 ± 30.56	29.64 ± 20.62	0.029
Gamma glutamyl transferase (GGT) (U/L)	32.30 ± 17.24	30.50 ± 17.41	0.284
Non-HDL-cholesterol	3.44 ± 0.73	3.44 ± 0.64	0.987
Fatty liver index	74.80 ± 18.57	69.01 ± 22.06	0.004

BMI: body mass index; EVOO: extra virgin olive oil; HbA1c: glycated hemoglobin; HDL: high density lipoprotein; LDL: low density lipoprotein; OC: oleocanthal.

**Table 2:** Effect of EVOO with a high OC concentration on plasma cytokines.

	Baseline	After 2 Months	*p*-Value
IL-6 (pg/mL)	2.9 ± 2.2	2.0 ± 1.9	0.020
IL-17A (pg/mL)	3.2 ± 4.0	1.5 ± 1.3	0.044
TNF-α (pg/mL)	7.3 ± 2.2	6.3 ± 2.1	0.003
IL-1B (pg/mL)	0.8 ± 1.4	0.2 ± 0.3	0.045
IL-10 (pg/mL)	0.4 ± 0.4	0.9 ± 1.3	0.039

EVOO: extra virgin olive oil; IL: interleukin; OC: oleocanthal; TNF-α: tumor necrosis factor alpha.

**Table 3:** Effect of EVOO with a high OC concentration on abdominal ultrasound parameters.

	Baseline	After 2 Months	*p*-Value
Visceral fat layer thickness (mm)	6.42 ± 2.23	6.32 ± 2.15	0.815
Min. subcutaneous fat thickness (mm)	1.18 ± 0.61	1.34 ± 0.51	0.170
Max. subcutaneous fat thickness (mm)	1.67 ± 0.74	2.19 ± 0.78	0.003
Min. pre-peritoneal fat thickness (mm)	0.56 ± 0.36	0.55 ± 0.23	0.633
Max. pre-peritoneal fat thickness (mm)	1.22 ± 0.82	1.11 ± 0.45	0.679
Ratio visceral fat layer thickness/max. subcutaneous fat thickness	3.98 ± 1.63	3.28 ± 1.49	0.020

EVOO: extra virgin olive oil; OC: oleocanthal.

**Table 4:** Baseline characteristics of all patients.

	All Cohort (*n* = 23)	Men (*n* = 15)	Women (*n* = 8)	*p* Value
Age (years)	60 ± 11	60 ± 13	59 ± 7	
Women, *n* (%)	8 (35)	0	8	<0.001
Smoking habit, *n* (%)	4 (17)	2 (13)	2 (25)	0.482
Family history of cardiovascular diseases, *n* (%)	15 (65)	10 (67)	5 (62)	0.842
Past history of cerebro-cardiovascular diseases, *n* (%)	0 (0)	0	0	
Hypertension, *n* (%)	14 (61)	9 (60)	5 (62)	0.907
Type-2 diabetes, *n* (%)	12 (52)	9 (60)	3 (37)	0.304
Dyslipidemia, *n* (%)	15 (65)	11 (73)	4 (50)	0.645
Obesity, *n* (%)	14 (61)	8 (53)	6 (75)	0.311
Use of anti-hypertensive therapies				
Aeta-blockers, *n* (%)	6 (26)	3 (20)	3 (37)	0.363
Angiotensin-converting enzyme inhibitors, *n* (%)	10 (43)	9 (60)	1 (12)	0.029
Calcium entry-blockers, *n* (%)	2 (9)	1 (7)	1 (12)	0.636
Diuretics, *n* (%)	4 (17)	4 (27)	0	0.108
Use of lipid-lowering drugs				
Statins, *n* (%)	8 (35)	6 (40)	2 (25)	0.472
Omega-3 fatty acids, *n* (%)	4 (17)	2 (13)	2 (25)	0.482
Ezetimibe, *n* (%)	2 (9)	2 (13)	0	0.280
Aspirin use, *n* (%)	9 (39)	8 (53)	1 (12)	0.056
Use of hypoglicemic drugs				
Biguanides, *n* (%)	10 (43)	8 (53)	2 (25)	0.192
Sulfonylureas, *n* (%)	2 (9)	2 (13)	0	0.280
Liraglutide, *n* (%)	1 (4)	1 (7)	0	0.455
